# Cost-Effectiveness of Enhanced Syphilis Screening among HIV-Positive Men Who Have Sex with Men: A Microsimulation Model

**DOI:** 10.1371/journal.pone.0101240

**Published:** 2014-07-01

**Authors:** Ashleigh R. Tuite, Ann N. Burchell, David N. Fisman

**Affiliations:** 1 Institute of Medical Science, University of Toronto, Toronto, Ontario, Canada; 2 Dalla Lana School of Public Health, University of Toronto, Toronto, Ontario, Canada; 3 Ontario HIV Treatment Network, Toronto, Ontario, Canada; 4 Faculty of Medicine, University of Toronto, Toronto, Ontario, Canada; UNC Project-China, China

## Abstract

**Background:**

Syphilis co-infection risk has increased substantially among HIV-infected men who have sex with men (MSM). Frequent screening for syphilis and treatment of men who test positive might be a practical means of controlling the risk of infection and disease sequelae in this population.

**Purpose:**

We evaluated the cost-effectiveness of strategies that increased the frequency and population coverage of syphilis screening in HIV-infected MSM receiving HIV care, relative to current standard of care.

**Methods:**

We developed a state-transition microsimulation model of syphilis natural history and medical care in HIV-infected MSM receiving care for HIV. We performed Monte Carlo simulations using input data derived from a large observational cohort in Ontario, Canada, and from published biomedical literature. Simulations compared usual care (57% of the population screened annually) to different combinations of more frequent (3- or 6-monthly) screening and higher coverage (100% screened). We estimated expected disease-specific outcomes, quality-adjusted survival, costs, and cost-effectiveness associated with each strategy from the perspective of a public health care payer.

**Results:**

Usual care was more costly and less effective than strategies with more frequent or higher coverage screening. Higher coverage strategies (with screening frequency of 3 or 6 months) were expected to be cost-effective based on usually cited willingness-to-pay thresholds. These findings were robust in the face of probabilistic sensitivity analyses, alternate cost-effectiveness thresholds, and alternate assumptions about duration of risk, program characteristics, and management of underlying HIV.

**Conclusions:**

We project that higher coverage and more frequent syphilis screening of HIV-infected MSM would be a highly cost-effective health intervention, with many potentially viable screening strategies projected to both save costs and improve health when compared to usual care. The baseline requirement for regular blood testing in this group (i.e., for viral load monitoring) makes intensification of syphilis screening appear readily practicable.

## Introduction

Major urban centers in the developed world have witnessed a dramatic re-emergence of syphilis in recent years, with the epidemic concentrated among men who have sex with men (MSM) and HIV-infected individuals [1]–[4]. Rates of syphilis acquisition among HIV-positive MSM up to 300-fold higher than those observed in the general male population have been reported [5]. Individuals who acquire syphilis may be asymptomatic, but untreated infection can lead to ocular, auditory, and neurological complications, even during the early stages of infection [6], [7]. By reducing the duration of infectiousness, early treatment also limits syphilis transmission to other individuals [8]. Canadian guidelines recommend that individuals at ongoing risk for syphilis be screened at 3-month intervals and that all MSM with STI risk, regardless of HIV status, undergo screening at least annually [9]. Annual syphilis testing rates were approximately 57% in HIV-infected MSM enrolled in the Ontario HIV Treatment Network Cohort Study (OCS) in 2009 [10].

The disproportionate and increasing burden of syphilis in MSM and HIV-infected individuals has led to calls for novel syphilis control strategies targeting these populations [11]–[13]. Men who are currently under medical care for HIV typically undergo routine blood work every 3 to 6 months [9]. Implementing routine syphilis serologic testing in this group presents a practical and inexpensive opportunity for increasing screening frequency, allowing for timely detection and treatment [14].

The current syphilis testing algorithm in Ontario, Canada includes a treponemal screening test followed by a confirmatory non-treponemal test (and a second confirmatory treponemal test). Although the treponemal test is more sensitive than the non-treponemal test for detecting early infection, once the test is positive, it often remains so for life, resulting in low specificity in individuals with previously treated syphilis [15], [16]. Follow-up non-treponemal tests can distinguish current from past (treated) infection, although with imperfect sensitivity and specificity. Increasing the frequency of syphilis screening in a population with high prevalence of previous syphilis infection, as is seen in HIV-positive MSM in Ontario [10], may thus result in a high number of false positives, leading to unnecessary treatment [17], [18] and diversion of limited public health resources [19].

Given the current burden of syphilis in HIV-positive MSM, it is unclear if the benefits associated with preventing new cases of neurosyphilis and tertiary syphilis via more frequent screening would outweigh the costs and health consequences associated with unnecessary treatment. Our objective was to evaluate whether strategies of enhanced (more frequent and/or higher coverage) routine syphilis screening in HIV-positive MSM receiving HIV medical care would be effective and economically attractive, relative to the current standard of care.

## Methods

### Model overview

We developed an individual-level state-transition (“microsimulation”) model [20] that follows simulated patients from time of model entry until death. The model included syphilis screening, the natural history of syphilis, and costs and consequences of syphilis screening and treated and untreated syphilis. We synthesized health and economic data related to syphilis in HIV-positive MSM; probability, cost, and quality-of-life estimates were derived from the published literature wherever possible and are presented in **Table 1**. The model was used to simulate 500,000 individual men similar to those enrolled in the Ontario HIV Treatment Network Cohort Study (OCS), an observational, open dynamic cohort of people receiving medical care for HIV infection in Ontario, Canada [21]. OCS data were used to estimate the proportion of men in the cohort with previous treated infection [10].

**Table 1:** Model variables and sources.

Variable	Details	Base Case Value	Range	Source
**Population characteristics**				
Age (yr, SD)		43.5	9.6	Burchell et al. [10]
CD4 count (cells/µL) (SD)		455	266	Burchell et al. [10]
Population with previous treated syphilis infection (%)^a^		21	–	Burchell et al. [10]
Annual incidence (%)	First infection	4.0	–	Burchell et al. [10]
	Re-infection	4.8	–	Burchell et al. [10]
Probability of death (per yr)	Baseline probability of death	Age-specific estimates		Statistics Canada [54]
	Excess mortality hazard in HIV-positive individuals	0.05	0.03–0.09	Bhaskaran et al. [25]
**Syphilis natural history**				
Average duration of syphilis stages (mo)	Primary	0.7	0.2–3.0	Garnett et al. [8]; PHAC [9]
	Secondary	3.6	0.5–6.0	Garnett et al. [8]; PHAC [9]
	Early latent	7.7	3.0–11.3	Garnett et al. [8]; PHAC [9]
Duration of immunity after treatment of late latent syphilis (yr)		5	1–10	Garnett et al. [8]
Time to develop late neurosyphilis (yr)		15	2–30	Golden et al. [44]
Probability of developing symptomatic neurosyphilis	Probability of neurologic involvement	0.33	0.2–0.4	Golden et al. [44]; Rolfs et al. [55]; Zetola and Klausner [25]
	Probability of developing early neurosyphilis^b^ (among those with neurological involvement)	0.05	0.03–0.09	Golden et al. [44]
	Probability of developing late neurosyphilis (among those with neurologic involvement)	0.09	0.04–0.14	Golden et al. [44]
Probability of recovery from symptomatic early neurosyphilis without disability, following treatment		0.70	0.54–0.83	CDC [22]
Odds of developing neurosyphilis if CD4 count <350 cells/mL (relative to CD4≥350)		3	1.3–7	Ghanem et al. [27]; Marra et al. [28]
Time to develop tertiary syphilis (yr)		20	10–30	PHAC [9]
Probability of developing tertiary syphilis (gummatous and cardiovascular)		0.25	0.15–0.35	Larsen [16]; Golden et al. [44]
**Test characteristics**				
Screening test (EIA) sensitivity	Primary	0.93	0.77–1	Sena et al. [15]
	Secondary	1	0.95–1	Owusu-Edusei et al. [18]; Sena et al. [15]
	Early latent	1	0.95–1	Owusu-Edusei et al. [18]; Sena et al. [15]
	Late latent	0.99	0.94–1	Owusu-Edusei et al. [18]
Screening test (EIA) specificity	No prior syphilis infection	0.99	0.94–1	Sena et al. [15]
	Previous treated syphilis infection	0.11	0.02–0.28	Blandford et al. [56]; Owusu-Edusei et al. [17]
Confirmatory test (RPR) sensitivity	Primary	0.86	0.77–0.99	Sena et al. [15]
	Secondary	0.99	0.95–1	Owusu-Edusei et al. [17]; Sena et al. [15]
	Early latent	0.98	0.95–1	Sena et al. [15]
	Late latent	0.73	0.37–0.94	Owusu-Edusei et al. [17]; Sena et al. [15]
Confirmatory test (RPR) specificity	No prior syphilis infection	0.98	0.93–0.99	Sena et al. [15]
	Previous treated syphilis infection	0.95	0.7–1	Owusu-Edusei et al. [17]
Probability of receiving lumbar puncture	Late latent, tertiary, neurosyphilis or any state with CD4 count ≤350 cells/µL	1		PHAC [9]
	CD4 count >350 cells/µL and not in late latent, tertiary or neurosyphilis stage	0		PHAC [9]
Probability of post-dural headache following lumbar puncture		0.2	0.06–0.36	Turnbull and Shepherd [57]
**Treatment characteristics**				
Probability of seeking treatment for syphilis symptoms	Primary	0.35	0.2–0.45	Bissessor et al. [14]; Kourbatova et al. [58]
	Secondary	0.6	0.4–0.85	Bissessor et al. [14]; Kourbatova et al. [58]
	Early latent	0.1	0.05–0.15	Bissessor et al. [14]; Kourbatova et al. [58]
Probability of treating individual identified as syphilis infected	True positive	0.95	0.8–1	Blandford et al. [59]; Assumption
	False positive, no prior history of syphilis infection	0.95	0.8–1	Blandford et al. [59]; Assumption
	False positive, history of treated syphilis infection	0.2	0–0.95	Assumption
Probability of treatment failure	Early syphilis^c^	0.05	0.02–0.09	Blank et al. [60]; Riedner et al. [61]
	Late syphilis	0.19	0.15–0.30	Blank et al. [60]; Riedner et al. [61]
Probability of anaphylaxis following treatment		0.0002	0.0001–0.0004	Tsevat et al. [62]
**HIV natural history and treatment**				
Set point viral load (log copies/mL)		4.6	–	Deeks et al. [63]; Mocroft et al. [64]; Rhone et al. [65]
Increase in CD4 count with initiation of ART (cells/µL)		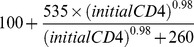	–	Drusano et al. [66]; Sanders et al. [30]
Decline in CD4 count with detectable viral load (cells/µL)		−79.2+33.5× log viral load	–	Cook et al. [67]
**Strategies**				
Achieved coverage	Higher coverage screening	1	–	Assumption
	Usual care	0.57	–	Burchell et al. [10]
**Costs (2011 CDN$)**				
Diagnostic tests (including labour)	Screening test	3.8	3.0–5.3	Public Health Ontario Laboratories (PHOL); Ontario Ministry of Health and Long-Term Care (MHLTC) [36]
	Confirmatory test	16.0	6.0–25.0	PHOL; Ontario Ministry of Health and Long-Term Care (MHLTC) [36]
	Lumbar puncture	275	128–309	PHOL; Ontario Ministry of Health and Long-Term Care (MHLTC) [36]
Treatment^d^	Early syphilis	400	200–600	MHLTC [35]; Assumption
	Late syphilis	635	320–950	MHLTC [35]; Assumption
	Neurosyphilis	14680	7340–22020	MHLTC [35]; Assumption
	Tertiary	4160	2080–6240	MHLTC [35]; Assumption
Adverse events^e^	Post-dural headache	66	33–99	Fisman et al. [68]; Assumption
	Anaphylaxis	4850	2425–7275	Fisman et al. [68]; Assumption
Lifetime cost of tertiary or neurosyphilis (excluding treatment)		91015	45005–136520	Owusu-Edusei et al. [38]
**Utilities**				
Base case, HIV-infected individual on ART		0.83	0.45–1	Sanders et al. [30]
Base case, HIV-infected individual, asymptomatic, not on ART		0.89	0.8–1	Sanders et al. [30]
Syphilis disutility	Primary syphilis	0.0072	0.0065–0.0079	Kwong et al. [31]; WHO [32]
	Secondary syphilis	0.041	0.036–0.045	Kwong et al. [31]; WHO [32]
	Neurosyphilis and tertiary syphilis (per year)	0.094	0.074–0.283	Kwong et al. [31]; WHO [32]
Lumbar puncture disutility	Procedure	0.01	0.005–0.05	Ward et al., [69]; Assumption
	Post-dural headache	0.02	0.005–0.05	Ward et al., [69]; Assumption
Disutility of treatment-associated anaphylaxis		0.02	0.007–0.03	Pepper and Owens [33]
**Other variables**				
Cycle length (mo)		1		Assumption
Discount rate (%)		5	0–5	CADTH [39]

a We assumed no prevalent infection at baseline.

b Early neurosyphilis refers to neurosyphilis that develops during the primary, secondary, or early latent stages of syphilis infection.

c Early syphilis refers to primary, secondary, or early latent syphilis infection.

d Base case treatment cost for neurosyphilis was calculated as the weighted average of inpatient and outpatient treatment, assuming 95% of cases require inpatient treatment. Base case treatment cost for tertiary syphilis was calculated as the weighted average of gummatous and cardiovascular syphilis, assuming 62% of tertiary syphilis cases are gummatous and 38% are cardiovascular [16]. Cardiovascular cases were further subdivided into those requiring surgery (20%) [70] and those not requiring surgery.

e Post-dural headache was assumed to require a general practitioner visit. Anaphylaxis was assumed to require hospitalization.

**Figure 1:**
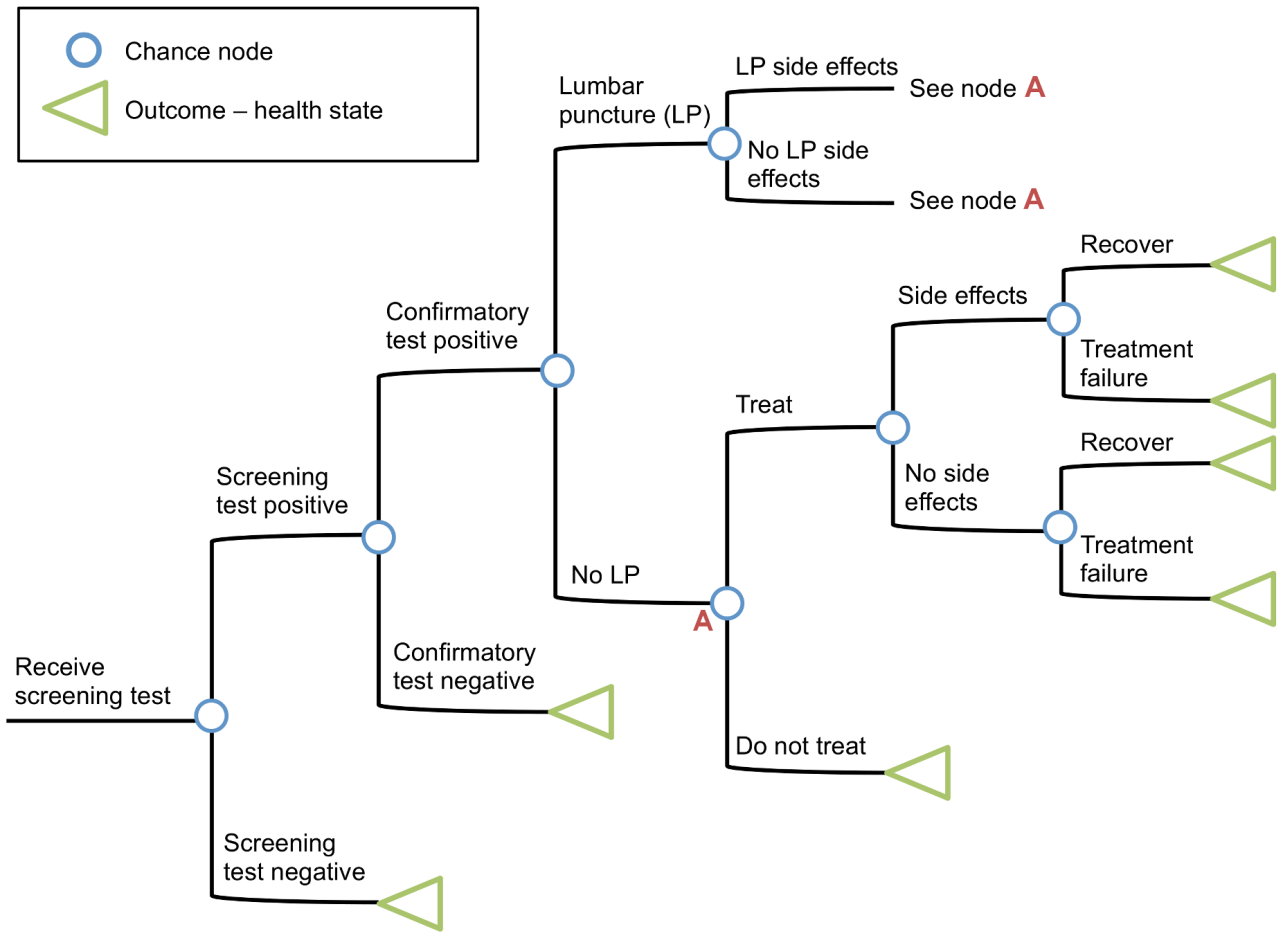
Simplified overview of screening and treatment component of the decision analytic model. A decision is made to screen or not screen HIV-positive MSM at risk of syphilis acquisition. If screening is performed, men can be correctly or incorrectly classified, resulting in appropriate or inappropriate use of resources, with associated costs and health consequences. Syphilis is treated according to the current Canadian guidelines. Chance nodes indicate points at which probabilities (described in **Table 1**) are applied. Individuals progress to the appropriate health state (outlined in **Figure 2**) following progression through the screening and treatment decision tree. Note that men who seek treatment for symptomatic syphilis infection follow the same set of decisions.

In our base case, we compared usual care to more frequent screening, and screening with higher population coverage. For the usual care strategy, 57% of the population received syphilis screening annually [10]. In the usual coverage – 3 and -6 months strategies, 57% of the population was screened every 3 or 6 months. In higher coverage strategies, 100% of the population was screened annually, or every 3 or 6 months. In all usual coverage strategies, individuals who had been screened once were more likely to be tested in the future [10]. Upon model initiation, 50% of individuals who were screened in the first round of screening were assigned to the regular screening group and were screened at all subsequent screening events; individuals in the non-regular screening group were selected at random at each screening event, such that the total proportion of the population screened equaled 57%. We assumed a standard screening algorithm consisting of a treponemal-specific assay (such as EIA) followed by a non-treponemal assay (such as RPR), which is now standard practice in Ontario. Test sensitivities were based on stage of infection, and specificities depended on prior infection history. After testing, men could be correctly or incorrectly classified as infected or uninfected, with incorrect classification resulting in the inappropriate use of resources (for false positives and false negatives). Among men with a history of previous treated syphilis, we assumed different probabilities of treatment for true vs. false positive cases, to capture the incorporation of additional information (such as reported sexual risk behavior) in a clinician's decision to treat. This assumption was tested in sensitivity analyses. Screening programs were conducted for 20 years in the base case. Each month, individuals could seek testing for syphilis symptoms. All individuals were eligible for screening, with frequency and uptake dependent on the strategy. We assumed that higher screening would be implemented as an opt-out policy with perfect compliance in our base case. We assumed that lumbar puncture was performed for all men diagnosed with late latent syphilis, neurosyphilis, or tertiary syphilis, and for those testing positive for syphilis with CD4 count<350 cells/mL [9]. An overview of the decisions involved in screening and treatment is presented in **Figure 1**.

**Figure 2:**
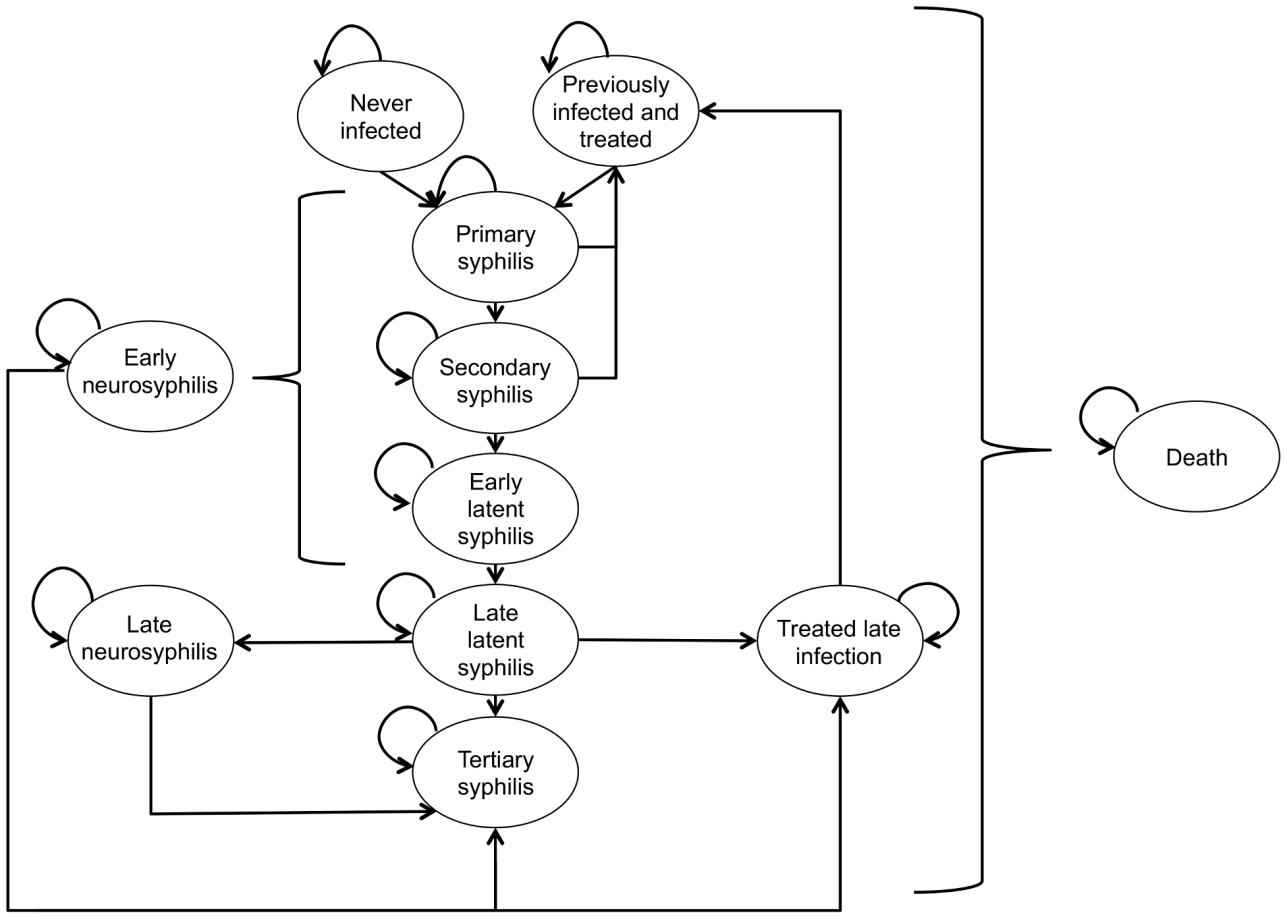
Markov model overview. The model has eleven health states, with allowed transitions between states indicated by arrows. Men have a chance of remaining uninfected or acquiring syphilis and progressing through the disease states. Syphilis infection is characterized by four stages: primary, secondary, early latent, and late latent, which may develop into tertiary syphilis. Men only exit the tertiary syphilis state via death, even if they receive treatment. Men may develop neurosyphilis at any stage of their syphilis infection. Men with early syphilis may receive treatment and recover without disability or have lifetime disability (indicated by entry into the tertiary syphilis state). All men with late neurosyphilis are assumed to have lifetime disability and enter the tertiary syphilis state. Men may transition to the death state from any model state. Treatment results in men returning to the ‘previously infected and treated' state. Movement through the model health states depends on transition probabilities identified from the literature. Disutilities are associated with the primary, secondary, tertiary, and neurosyphilis states and long-term healthcare costs are associated with tertiary and neurosyphilis.

Our Markov model consisted of mutually exclusive and collectively exhaustive health states: uninfected; primary syphilis, secondary syphilis and early latent syphilis (“early syphilis”); late latent syphilis; neurosyphilis; tertiary syphilis; treated late syphilis; and death (**Figure 2**). The uninfected state was subdivided into uninfected with no prior syphilis infection and uninfected with previously treated syphilis infection, to capture different syphilis incidence [10], test specificities and probabilities of treatment following positive test results in these two groups. The neurosyphilis state was divided into early symptomatic neurosyphilis and late neurosyphilis, to reflect the fact that individuals treated for early neurosyphilis could recover without long-term disability [22].

In the base case, incidence of syphilis infection was based on estimated rates observed in the OCS for MSM, with infection rates higher in men with prior syphilis [10]. As surveillance data suggest that risk of syphilis infection is low in individuals aged ≥65 [4], we assumed that men were at risk of syphilis acquisition for a period of 20 years after model entry, although this was varied in sensitivity analyses. Monthly transitions between stages of infection (i.e., primary to secondary, secondary to early latent, early latent to late latent) and return to the uninfected state following treatment for late syphilis, were derived from mean estimates of time spent in each stage and were converted to probabilities assuming an exponential distribution [23]. Individuals treated for primary, secondary, or early latent syphilis were assumed to return immediately to the uninfected state. Individuals successfully treated for late latent or early symptomatic neurosyphilis with no long-term disability entered a transient immune state prior to returning to the uninfected state [8]. Individuals with early symptomatic neurosyphilis were assumed to either recover after six months, or transition to the tertiary syphilis state, to capture the long-term health consequences associated with their health state. All individuals with late neurosyphilis were assumed to have long-term health consequences and transitioned to the tertiary state following treatment, to capture the associated lifetime costs and disutility. Tertiary syphilis was considered to be associated with long-term sequelae whether or not treatment was received (i.e., transitions back to morbidity-free health states were disallowed). Mortality rates were based on Canadian life tables [24], the excess mortality observed in HIV-infected individuals compared to the general population [25], and the excess mortality associated with untreated tertiary syphilis [26].

In all strategies, syphilis infection was managed according to the current Canadian guidelines with early syphilis treated with a single dose of benzathine penicillin, late syphilis treated with three once-weekly doses, and neurosyphilis treated with high dose intravenous penicillin [9]. To account for enhanced risk of neurosyphilis with lower CD4 counts [27], [28] and CD4 count-based recommendations for lumbar puncture upon syphilis diagnosis [9], we modeled progression of HIV infection, as measured by changes in viral load and CD4 level. Antiretroviral therapy (ART) was initiated when CD4 count was ≤500 cells/mL [29]. We did not model interruptions in ART. In the absence of ART, men were assumed to experience a decline in CD4 count. Health outcomes measured intermediate disease-specific outcomes and changes in quality adjusted survival. We used previously published estimates of the quality-of-life of individuals with HIV [30], disutility associated with primary, secondary, tertiary, and neurosyphilis, and the adverse outcomes associated with lumbar puncture and treatment [31]–[34].

### Costs

Costs are presented in **Table 1**, and included testing, treatment and follow-up costs, as well as lifetime costs for untreated individuals who progressed to tertiary disease. We used Ontario-specific testing and treatment costs [35], [36]. Treatment costs depended on disease stage and included medication and physician costs, and costs of follow-up visits and testing, with the number of follow-up tests based on Canadian treatment guidelines [9]. Adverse event costs included a general practitioner visit for post-dural headache and hospitalization for anaphylaxis. All costs were converted to year 2011 Canadian dollars using the health and personal care component of the Canadian Consumer Price Index [37]. In the absence of Canadian data about lifetime costs associated with tertiary syphilis, we used costs derived from the United States [38], which were adjusted for inflation and converted to 2011 Canadian dollars.

We performed cost-effectiveness analyses from a public Canadian health care payer perspective, which approximates a societal perspective in a country like Canada with publicly funded health services, but ignores patient time and travel costs. Cost effectiveness of each strategy was estimated as the incremental cost per QALY gained relative to the next most expensive strategy. Since the effects of untreated syphilis often takes decades to manifest, we used a lifetime time horizon. A base case discount rate of 5% was applied to future costs and outcomes [39].

### Model Validation and Simulations

To ensure that our model was reproducing observed trends in reported syphilis infection, we compared the expected incidence of diagnosed early neurosyphilis in our modeled cohort under the usual care scenario to estimated rates of neurosyphilis between 2008 and 2012 [4] in Toronto's HIV infected male population [40]. Our base case analysis was performed as a first-order Monte Carlo simulation of 500,000 individuals assigned to each strategy. To account for uncertainty surrounding parameter estimates we performed probabilistic sensitivity analyses using second-order Monte Carlo simulations, such that parameters were sampled from distributions for each of 1000 simulated trials; each trial included 1000 identical “individuals” with costs and outcomes projected in stochastic (first-order) simulations. We used gamma distributions for costs and beta distributions for probabilities and utilities [41], with plausible ranges based on 95% confidence intervals, lowest and highest published values, or by varying inputs by ±50%, depending on available data. We performed additional sensitivity analyses with alternate assumptions around treatment probabilities, syphilis incidence, infection risk period, program duration, compliance with higher coverage strategies, and CD4 count at initiation of ART.

## Results

### Model Validation and Projected Effectiveness

**Figure 3:**
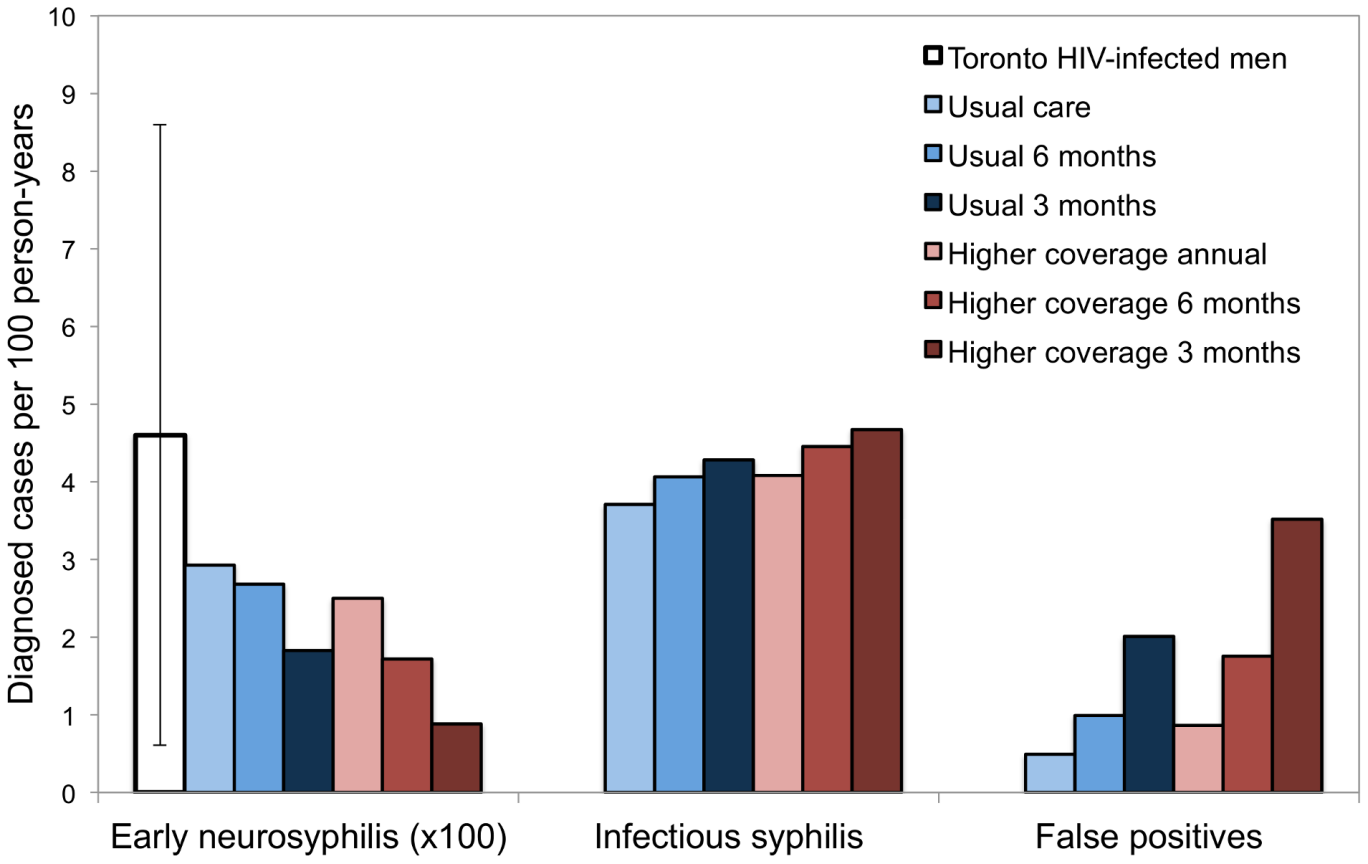
Model validation and projections. Model estimated diagnosis of early neurosyphilis, infectious (primary, secondary, and early latent) syphilis, and false positive cases. Reported values represent the average rates in the modeled cohort over a 20-year period for the different strategies evaluated. Neurosyphilis infections are plotted x100 for comparability. Usual care – annual represents model projections based on current estimates of screening coverage and frequency among HIV-infected MSM under medical care. Toronto HIV-infected men represents estimated rates of diagnosed early neurosyphilis among HIV infected men living in Toronto (average for the years 2008–2012, error bars represent 95% confidence intervals). Usual care, usual 6 months, and usual 3 months refer to screening 57% of the population every 12, 6, or 3 months, respectively. Higher coverage annual, 6, months and 3 months refer to screening 100% of the population every 12, 6, or 3 months, respectively.

In the absence of enhanced (more frequent and/or higher coverage) screening, approximately 2.9 per 10,000 men were projected to be diagnosed with neurosyphilis annually, which is within the range of rates of 0.7–9.1 (mean 4.5) per 10,000 HIV-infected males in Toronto between 2008–2012. Compared to usual care, higher (100%) coverage 3-monthly screening was projected to reduce incidence of diagnosed early neurosyphilis (0.9 per 10,000 person-years), while increasing the diagnosis of infectious syphilis (from 3.7 to 4.7 per 100 person-years). Unnecessary treatment of uninfected men due to false positive test results also increased (from 0.5 to 3.5 per 100 person-years) (**Figure 3**). The additional strategies resulted in infection incidence intermediate between usual care and higher coverage 3-monthly strategies.

### Cost Effectiveness

Compared to the usual care (57% coverage, annual) strategy, higher (100%) coverage 3-monthly screening was projected to cost more ($98.69) and be more effective (0.015 QALY) (**Table 2**). All intermediate strategies cost less than the usual care strategy, but provided fewer QALY gains than higher coverage 3-monthly screening. The differences in QALYs between strategies were minimal, reflecting the fact that most health consequences of latent syphilis occur years or decades after initial infection.

**Table 2:** Discounted health and economic outcomes associated with different syphilis screening strategies.

Strategy^a^	Discounted Cost^b^ (CDN $)	Incremental Cost (CDN $)	Discounted Life Expectancy (y)	Discounted Effectiveness^b^ (QALY)	Incremental Effectiveness (QALY)	ICER ($/QALY)
Higher coverage, 6 months	1019.51	–	16.0871	13.3497	–	–
Higher coverage, annual	1059.74	40.22	16.0888	13.3468	−0.0030	Dominated
Usual, 6 months	1148.20	128.69	16.0891	13.3466	−0.0031	Dominated
Usual, 3 months	1195.81	176.30	16.0824	13.3448	−0.0049	Dominated
Usual care	1310.25	290.73	16.0855	13.3398	−0.0099	Dominated
Higher coverage, 3 months	1408.94	389.42	16.0892	13.3548	0.0050	77,516.35

Abbreviations: QALY, quality-adjusted life year; ICER, incremental cost-effectiveness ratio.

a Higher coverage, 100% coverage; Usual, 57% coverage.

b Discounted at 5%.

**Figure 4:**
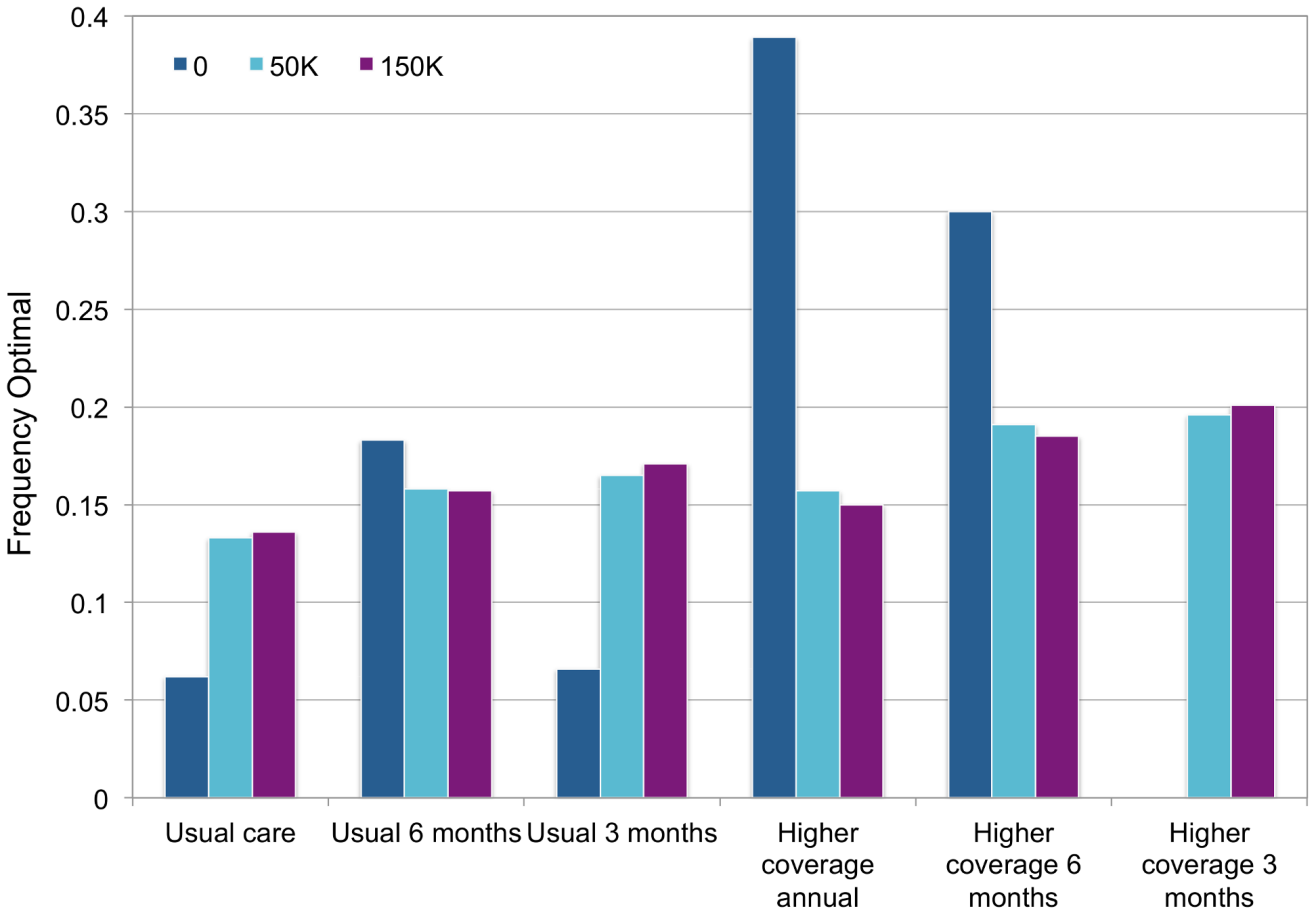
Strategy acceptability for different willingness-to-pay thresholds. The frequency with which each strategy was optimal at willingness-to-pay thresholds of 0, $50,000, or $150,000 per QALY is shown for 1000 probabilistic trials with 1000 individuals assigned to each strategy within each trial. Usual care, usual 6 months, and usual 3 months refer to screening 57% of the population every 12, 6, or 3 months, respectively. Higher coverage annual, 6, months and 3 months refer to screening 100% of the population every 12, 6, or 3 months, respectively.

To capture second-order uncertainty, we performed 1000 simulated trials, each with 1000 participants randomly assigned to each screening strategy. The preferred strategy varied depended on the willingness-to-pay threshold (**Figure 4**). Assuming a willingness-to-pay (WTP) of $0 per QALY, higher coverage screening every 3 or 6 months was most frequently preferred. At a WTP threshold of $50,000 or $150,000/QALY, preferences for individual strategies were less clear, though in general higher coverage strategies were preferred to lower coverage strategies of the same frequency, and there was an approximately linear relationship between increased screening frequency and likelihood that a strategy would be preferred.

### Sensitivity Analysis

As uncertainty in model parameters was incorporated into second order Monte Carlo simulations, we performed additional sensitivity analyses using alternate assumptions about disease epidemiology, natural history and programmatic features (**Table 3**). As we reduced syphilis risk, we found that usual care became a viable strategy when risk of infection declined to<20% of baseline risk, and even in this circumstance, higher coverage 6-monthly screening remained a highly cost-effective strategy ($39,096 per QALY). We identified no other circumstance under which usual care was a non-dominated strategy. Similarly, usual screening coverage strategies with increased frequency of screening seldom emerged as viable strategies. We found that usual coverage screening at a frequency of every six months emerged as a potentially viable strategy only when the probability of treatment of false-positive tests became equivalent to the probability of treating true-positive syphilis (ICER for usual coverage screening every 6 months $13,807 per QALY relative to higher coverage annual screening).

**Table 3:** Preferred syphilis screening strategies under alternate model assumptions and for different willingness-to-pay thresholds.

		Preferred Strategy^a^ by Willingness-to-Pay Threshold ($/QALY)		
Variable	Details	0	50,000	150,000
Base case^b^		Higher coverage 6	Higher coverage 6	Higher coverage 3
Probability of treating false positive cases among previously infected men	0	Higher coverage 6	Higher coverage annual	Higher coverage 3
	0.5	Higher coverage annual	Higher coverage annual	Higher coverage annual
	0.95	Higher coverage annual	Usual 6	Higher coverage 3
Probability of treating false and true positive cases among previously infected men	0.2	Higher coverage 3	Higher coverage 3	Higher coverage 3
	0.5	Higher coverage 6	Higher coverage 3	Higher coverage 3
	0.75	Higher coverage annual	Higher coverage 6	Higher coverage 6
Syphilis incidence	2-fold increase	Higher coverage 6	Higher coverage 6	Higher coverage 6
	5-fold increase	Higher coverage 3	Higher coverage 3	Higher coverage 3
	2-fold decrease	Higher coverage annual	Higher coverage 6	Higher coverage 6
	5-fold decrease	Usual care	Higher coverage 6	Higher coverage 6
	Linear decrease from current level to 0 over 20 years	Higher coverage annual	Higher coverage annual	Higher coverage annual
CD4 count at which initiate ART	350 cells/mL	Higher coverage 6	Higher coverage 6	Higher coverage 3
Uptake of higher coverage screening	60–90%	Higher coverage 6	Higher coverage 6	Higher coverage 6
Duration of infection risk/duration of screening program (yr)	10/10	Higher coverage 6	Higher coverage 6	Higher coverage 6
	10/20	Higher coverage annual	Higher coverage 6	Higher coverage 6
	10/30	Higher coverage annual	Higher coverage 6	Higher coverage 6
	20/30	Higher coverage annual	Higher coverage 6	Higher coverage 6
	30/30	Higher coverage 6	Higher coverage 6	Higher coverage 6

a Higher coverage annual, 6, and 3 refer to screening 100% of the population every 12, 6, or 3 months, respectively (except for the analysis where uptake was varied from 60–90%). Usual care and usual 6 refer to screening 57% of the population every 12 or 6 months, respectively.

b In the base case, probability of treating a false positive case with prior history of syphilis infection was 0.2; probability of treating a false positive case with no prior history of syphilis infection and all true positive cases was 0.95; syphilis incidence was 4 per 100 person-years (py) in never infected men and 4.8 per 100 py in previously infected men; ART was initiated when CD4 count was <500 cells/mL; uptake of higher coverage screening was 100%; and duration of infection risk and duration of screening program were both 20 years.

In other sensitivity analyses, higher coverage strategies dominated usual coverage strategies (i.e., cost less and provided greater health benefit) but the optimal screening interval was sensitive to input parameters. In our base case we assumed that treatment with combination ART would begin when CD4 counts reached 500 cells/mL; when we assumed ART at a CD4 count of 350 cells/mL, higher coverage every 3 months became costly relative to every 6 months (ICER $130,834 per QALY) but might still be considered cost-effective in high income countries [42]. Higher coverage every 6 months was the preferred strategy when duration of infection risk was varied from 10 to 30 years (assuming a WTP of $50,000/QALY), regardless of whether screening was halted once syphilis acquisition risk stopped or continued beyond the infection risk period. When we assumed equal probability of treatment of true and false positive cases among men with a history of previous treated syphilis, higher coverage was the preferred strategy, with frequency of screening depending on the assumed level of treatment. Decreasing coverage in the higher coverage strategies to 60–90% resulted in higher coverage every 6 months dominating all of the other strategies. When costs and health outcomes were not discounted, higher coverage every 6-months dominated all other strategies except for 3-monthly higher coverage (ICER $239,539 per QALY) (**Table 4**).

**Table 4:** Undiscounted health and economic outcomes associated with different syphilis screening strategies.

Strategy^a^	Cost (CDN $)	Incremental Cost (CDN $)	Life Expectancy (y)	Effectiveness (QALY)	Incremental Effectiveness (QALY)	ICER ($/QALY)
Higher coverage, 6 months	1661.30		35.1304	29.129		
Higher coverage, annual	1834.26	172.96	35.1079	29.1241	−0.005	Dominated
Usual, 3 months	1959.64	298.34	35.0891	29.1037	−0.0253	Dominated
Usual, 6 months	2003.24	341.94	35.1271	29.1252	−0.0039	Dominated
Higher coverage, 3 months	2225.39	564.08	35.1205	29.1314	0.0024	239,539
Usual care	2499.95	838.65	35.1077	29.0968	−0.0323	Dominated

Abbreviations: QALY, quality-adjusted life year; ICER, incremental cost-effectiveness ratio.

a Higher coverage, 100% coverage; Usual, 57% coverage.

## Discussion

We project that a universal syphilis screening program in HIV-infected MSM under medical care has the potential to improve health and save costs, relative to usual care. However, increased screening may result in unnecessary treatment of men with false positive tests and lead to excess adverse events associated with testing and treatment (e.g., adverse consequences of lumbar puncture). Although previous studies have examined the disease dynamic impacts of screening frequency, [11], [43] with projections that more frequent screening would reduce syphilis burden in MSM, to our knowledge, this model is the first to evaluate the cost-effectiveness of more frequent and higher coverage syphilis screening in a high-risk group.

Perhaps most importantly, we project that both increases in test frequency and coverage for MSM with HIV infection would increase effectiveness relative to current standard of care, and that all more-intense screening regimens (with the exception of higher coverage, 3-monthly screening) would decrease, rather than increase, net healthcare costs due to aversion of downstream sequelae of untreated syphilis infections. Although the projected increase in effectiveness observed with enhanced (higher coverage and/or increased frequency) screening was small compared to usual care, this reflects the fact that the major health consequences of syphilis occur years, and often decades, after initial infection [8], [44], and consequently the health impact of screening is reduced by discounting.

We restricted our analysis to HIV-positive MSM because surveillance data show this to be the population at greatest risk of syphilis in much of North America [45]–[47]. Men currently under medical care for HIV present an ideal target for this intervention, since syphilis screening can be included with existing blood-work with minimal inconvenience and expense [14]. By implementing an opt-out syphilis screening test, we would expect screening frequency in HIV-positive MSM to increase [14]. The major concern with implementing such a strategy is the management of false positive test results in individuals with a history of previously treated syphilis infection and the resulting physician burden [48]. With more frequent RPR testing, clinicians will have a better history of an individual's titre, assisting with the interpretation of titre changes and helping to distinguish new infections from past cases (in particular serofast cases). Appropriate management of individuals with previous treated syphilis infection would be crucial for minimizing unnecessary treatment and the development of clear guidelines about how to manage test results would be critical.

Our study is subject to several limitations. Our estimates of syphilis acquisition risk are based on HIV-positive MSM in Ontario [10], our target population, and are somewhat higher than reported estimates in MSM in other jurisdictions [45], [49], [50]. Nonetheless, we found our projections of cost-effectiveness to be robust in the face of variation in syphilis risk, and elevated syphilis risk in MSM is currently widespread in North America and Europe [4], [51]. It is important to note that syphilis epidemics tend to occur in waves separated by periods of low incidence, and during the latter periods the screening strategies we identify as cost-effective in this analysis may cease to be attractive. As discussed above, interpretation of titre changes is important for individual case management; we did not include treatment algorithms for serofast cases in the model, due to the associated complexity. Our model included simplifying assumptions and incorporates parameter values that are subject to uncertainty, but our findings were robust in the face of wide-ranging sensitivity analyses and alternate assumptions.

Lastly, we used a traditional static risk Markov model to evaluate the health-economic attractiveness of syphilis screening in MSM. Such models are limited in their ability to capture the indirect effects of disease treatment and prevention that are characteristic of communicable disease control programs [52], including not only reductions in the future stream of syphilis infections but also the impact of untreated syphilis infections on the transmission of HIV [53]. However, our previously published work on screening effectiveness in a dynamic, agent-based model [43] similarly identified an increase in effectiveness of screening as a means of reducing syphilis incidence with increased frequency of syphilis screening. Work remains for the quantification of the economic attractiveness of syphilis screening strategies in a manner that includes both direct and indirect effects of screening.

In summary, we have shown that when rates of syphilis acquisition are high, implementing routine syphilis screening in MSM currently under medical care for HIV is expected to be a highly cost-effective intervention. This strategy has been implemented in Australia and has been demonstrated to increase the detection of early asymptomatic syphilis [14], but the long-term effectiveness and cost-effectiveness of this intervention has not been evaluated. While a clinical trial would be an ideal means of testing our model projections, the policy-relevant time horizon would likely exceed the attainable duration of such a trial. Our model provides an estimation of the potential impact of an enhanced syphilis screening program among HIV-positive MSM that can help guide policy decisions.
